# Indian contribution to behavior therapy

**DOI:** 10.4103/0019-5545.69271

**Published:** 2010-01

**Authors:** K. Kuruvilla

**Affiliations:** Department of Psychiatry, PSG Institute of Medical Sciences and Research, Coimbatore, India

**Keywords:** Behavior therapy, behavior modification, behavioral intervention, cognitive therapy, cognitive behavior therapy

## Abstract

Publication of papers related to psycho-social interventions in general and Behavior Therapy, in particular, in Indian Journal of Psychiatry has been limited. Though the first paper related to Behavior Therapy was published in 1952, a manual search of all available issues of the journal from 1949 showed that only 42 papers related to Behavior Therapy have been published till 2009. Among them 10 are case reports. Methodological limitations abound even in the papers on larger groups of patients. Studies using operant conditioning have been very few. Aversion therapy and progressive muscle relaxation have been very frequently used. The published articles are reviewed under the various diagnostic categories. Publications in the recent years have been mostly on Cognitive Behavior Therapy. Even after 57 years of co-existence, the relationship between Behavior Therapy and Indian Psychiatry remains a tenuous one.

## INTRODUCTION

One of the characteristics of psychiatry, as practised in India, is the extremely limited use of psycho-social methods of treatment. In 1973, when Varma and Ghosh[1] did a survey on the practice of psychotherapy among Fellows of Indian Psychiatric Society, only 17% of them reported to be using any psychological method of treatment. Today we have a much larger number of psychiatrists in this country, but the percentage of those using psychological methods of treatment is unlikely to be much greater. This limited use of psychological method in clinical practice (even in academic centers) is reflected in the publications in this area. It is found that among the papers published in Indian Journal of Psychiatry (IJP) only about 2% deal with psycho-social methods of treatment, in comparison to 16% in the British Journal of Psychiatry.

The above observation on psychological methods of treatment in general is also true with regard to Behavior Therapy (BT) or any of its variants. Most psychiatrists in India, I feel, will admit that these psychological methods are useful or even essential in some psychiatric disorders, but many of them do not have either the time or inclination to practice them. Often such patients are referred to psychologists or social workers who are interested in psycho-social methods of treatment. So it is likely that publications like Indian Journal of Clinical Psychology may have many more papers on BT than Indian Journal of Psychiatry. However, the present article is not an attempt to review the state of BT in India, but is a more restricted attempt to review articles published in Indian Journal of Psychiatry since it will more accurately reflect the Indian psychiatrists’ attitude towards BT.

### Method of search for articles

Using the key words ‘Behavior Therapy’ on the IJP web site yielded a list of 42 articles related to the topic. However, when one went through the list only nine articles were found to have anything to do with BT and the rest were on totally unrelated topics ranging from ‘ECT and clozapine’ to ‘psychotic symptoms in acromegaly’!

So a manual search was done through the contents of all available issues of Indian Journal of Neurology and Psychiatry (1949 to 1953) and Indian Journal of Psychiatry (1958 to 2008) in the DVD published by Indian Psychiatric Society. Contents of the three issues of the Journal published in 2009 were also searched. Articles which contain words like - Behavior therapy, behavior modification, behavioral intervention, cognitive therapy, and cognitive behavior therapy, biofeedback, relaxation training, which are related to BT, were searched. In addition, if the title of the articles suggested the possibility of BT use, for example ‘Treatment of Trichotillomania,’ then the articles were given a detailed look to see whether the authors have in fact used BT and those papers were also selected.

### In the beginning

The article which could be considered the first on this topic was authored by Ganguly[2] in Indian Journal of Neurology and Psychiatry in the year 1952. It was entitled “Pavlov’s Influence on Psychiatry” and discusses ‘Pavlovian Reflexology’ and introduces the concepts of conditioned reflex, experimental neurosis etc. and suggests that these concepts can help psychiatrists in understanding and treating disorders like hysteria, obsession, paranoia and psychosomatic disorders. The author reported that he was working on asthma and peptic ulcer on Pavlovian lines and expressed the hope to publish the results at a future date. However, we do not have any information on the fulfillment of this promise.

It may come as a surprise to many readers, especially of the older generation, that Satyanand[3] who has written extensively on psychoanalytically oriented psychotherapy in the Indian context, also has written an article on ‘Recent Advances in Behavior Therapy’. Initially the paper deals with psychotherapy in general and the need to use it in psychiatric practice and then introduces BT which at that time was not a well known system of therapy. The author concludes: “If the medical man wants to work out psychotherapeutic sessions on his own, he will find it easier to follow the Behavior Therapy and Reconditioning Therapy”.

Skepticism about the newly emerging treatment method is evident in Neki’s[4] review of a book called ‘Behavior Therapy in the 1970s’. He writes, “broader basic principles, more complex psychopathological models and therapeutic procedures with wider range of effectiveness need yet to be developed and social learning, cognitive mediating mechanisms related to attitudes, value systems and self concepts need to be incorporated in to the practice of behavior therapy”. This sagacious advice became a reality in the next decade with the advent of Cognitive Therapy (CT) and the fusion of cognitive therapy and behavior therapy in to Cognitive Behavior Therapy (CBT).

In the early 1970s BT was still a new entrant in to the field of psychiatry in India. This is evident from the two articles by Chopra.[5,6] In the first one, Jacobson’s Progressive Muscle Relaxation (JPMR) is introduced to Indian readers as “treatment for neurotic states in which anxiety and phobic features are predominant” and also mentions it as a part of ‘systematic desensitization’. The technique is illustrated through case reports. In the second article, treatment of 11 patients with disorders like agoraphobia, obsessive compulsive disorder and frigidity with systematic desensitization is described.

All 11 patients improved (six markedly improved) with the treatment. On follow-up varying from one month to two years only one patient was found to have relapsed. Though published in IJP, the papers were based on the author’s work in Australia. It is interesting to note that Chopra as well as other authors at that time, while talking about outcome on follow-up usually added “there was no symptom substitution,” perhaps to counter the criticism of psychoanalytically oriented therapists who held that BT is only removing symptoms at a superficial level and since the deep seated conflicts are not dealt with, the patient will invariably present with some other symptoms at a later date.

### General characteristics of behavior therapy articles published in IJP

1. BT was the first psychological method of treatment which showed that its therapeutic efficacy can be established by rigorous scientific methods of evaluation like randomized, placebo-controlled, double blind studies, instead of merely relying on the subjective opinions of patients and therapists. It has shown that methodological issues like sample size, randomization, use of controls, masking of the assessor, valid outcome measures, determination of statistical as well as clinical significance of outcome etc. are important and feasible in evaluating efficacy of psychotherapeutic methods also. But when we go through the published studies in India it becomes obvious that we have not paid attention to such methodological issues.
2. Out of the 42 articles related to BT till 2009, 10 are single case reports. In a newly emerging model of treatment single case reports have a place. BT papers by western workers have shown that efficacy of treatment technique can be more convincingly demonstrated, even in a single case by following an A-B-A-B design. But none of our case reports have included such a step.
3. Main model of BT as practiced in India seems to be classical conditioning. Operant conditioning which has emerged as a more versatile and effective approach and has a greater degree of acceptance by patients, has few adherents here.
4. Excessive reliance on aversive techniques is seen in many of the studies in a wide variety of disorders-hysteria, homosexuality, tremors, titubation etc. It is interesting to note that even in the 1970s, in the ‘Instructions to Authors” in the well known BT journal, ‘Behavior Therapy and Experimental Psychiatry’ (edited by Joseph Wolpe), it was stated that reports using aversion therapy techniques will not be accepted, unless no other proven method of treatment exists for that condition.
5. Jacobson’s Progressive Muscle Relaxation (JPMR) is used as a part of BT in almost all conditions ranging from nail-biting to schizophrenia! Justification for its use is not given in many of the papers, so much so, even in some of our academic institutions JPMR is considered to be synonymous with BT, even to the extent of postgraduate trainees in psychiatry who are posted in Behavior Therapy units getting trained only in JPMR.
6. Many of the reports show that every behavioral technique described in a standard text book on the subject is used for every disorder, often without justifying such a multipronged approach, which reduces the cost effectiveness of the therapy and its application in settings with limited time and trained personnel.

### Behavior therapy in specific disorders

#### Sexual dysfunction

Agarwal’s[7] paper in 1970 described the treatment of erectile dysfunction (ED) and premature ejaculation (PE). Treatment consisted of sex education, assessment of marital relationship and ‘deconditioning’ which resulted in improvement in nine out of the 11 patients. Though the author does not use the term BT and emphasizes the psychotherapeutic aspects of the treatment in the discussion, the procedure described as ‘deconditioning of the faulty ejaculation response’ is very similar to behavioral techniques used in sex therapy. Behavioral techniques described by Wolpe were used by Kuruvilla[8] in the treatment of 18 men with SD and 8 with SD and PE. All were treated along with their wives by a single therapist. At the end of the treatment, 54% were much improved, and others were partially improved, as reported by patients and their wives independently. At follow-up all but one of the ‘much improved’ persons maintained the improvement. Bagadia *et al*.[9] treated 26 patients with PE and secondary ED with BT. Wives of only 11 patients attended therapy sessions. Yet 58% of patients improved. The authors opine that wives not coming for treatment sessions does not necessarily indicate marital disharmony in most of the patients and a wife who is unable to attend the sessions may still help in the treatment by following instructions sent through the husband. Gupta *et al*.[10] treated 21 married couples belonging to urban middle class and engaged in white collar jobs, with what the authors called as “modified Masters and Johnson technique” because only one therapist was involved and psychoanalytical principles also were used. 76.2% were reported to have “recovered”. The authors attribute the better outcome, in comparison to other studies, to the use of psychoanalytical concepts in addition to BT. But other factors like difference in sample characteristics also could have led to the difference in outcome.

In an attempt to help unmarried men who present with ED, but were unable to go through the classical Masters and Johnson sex therapy, because of the lack of a partner, Kuruvilla[11] introduced the technique of guided imagery and masturbatory conditioning with the help of erotic reading material. Relaxation training and sex education were given prior to these steps to reduce the sexual misconceptions and associated high anxiety level. Among the 18 patients without a partner who were offered this treatment, 13 completed the course. Response was rated as ‘good’ in nine and ‘partial’ in four. Eleven of the completers could be followed up for six months to two years and at the time of last follow-up contact seven of them were completely free from the erectile problem.

The above reviewed studies highlight some of the modifications introduced by Indian therapists to deal with certain special difficulties encountered in our country, in the treatment of sexual dysfunctions.

### Disorders of sexual preference

In the 1970s and 80s, men with ego-dystonic homosexuality often came to Indian psychiatrists for help and behavioral techniques were used in treating such persons. Sakthivel *et al*.[12] treated four men who voluntarily approached them for treatment to get rid of their homosexual orientation. They were treated with anticipatory avoidance technique. These men also experienced high anxiety levels when they had to interact with females. This was treated by desensitization. They were followed up from 5 to 10 months. At follow-up, all reported being free from homosexual behavior and having heterosexual interests. One was happily married. 61% of the13 males with homosexual orientation treated by Pradhan *et al*.[13] with electrical aversion for homosexual imagery and positive conditioning for heterosexual imagery, reported change in sexual orientation. Patients’ motivation to change was found to be the main factor associated with good outcome. Mehta *et al*.[14] treated six persons with homosexual orientation with ‘double differential conditioning,’ which consisted of pairing Faradic electrical aversive stimuli with homosexual fantasy and music with heterosexual fantasy. Social skills training and supportive psychotherapy also were given. Four of the treated individuals achieved a change in sexual orientation.

A case of frotteurism was treated by Kuruvilla *et al*.[15] with systematic desensitization in imagery and *in-vivo* for eliminating the avoidance behavior to social situations which produced anxiety in him because of frotteurism. Frotteurism itself was treated with exposure to situations of bodily contact with males in imagery and *in-vivo* and also aversion therapy. Symptoms were eliminated in 10 sessions. This patient was followed up for one year when he was found to have no anxiety in social situations and no indulgence in frotteurism. BT consisting of relaxation training, aversion therapy with aversion relief, modeling, orgasmic reconditioning, behavioral counseling and sex education, was used by Andrade *et al*.[16] for a patient with trans-sexualism and homosexual orientation. Therapy resulted in normalization of gender identity, but homosexual orientation persisted.

### Obsessive compulsive disorder

Nammalvar *et al*.[17] treated 17 patients with a diagnosis of obsessive compulsive disorder. In the first phase, they were taught progressive muscle relaxation, to be practiced at home regularly. Thought stopping was introduced in the second phase. Therapeutic change was measured using Taylor’s Manifest Anxiety Scale and Beck Depression Inventory before and after each phase. 65% of the patients showed marked reduction in the frequency of obsessions and the distress caused by them. There was also significant reduction in anxiety and depression. Good outcome was found to be associated with short duration of illness and low level of depression. On follow-up, ranging from one to four years, 10 out of the 11 who showed improvement were found to maintain the improvement. The paper does not give any information on the effect of treatment on the compulsive symptoms some of these patients had or whether some other treatment method was used to deal with them. In another paper on the same group of patients, these authors[18] suggest the phenomenon of habituation as the explanation for the effectiveness of thought stopping. In the absence of empirical data this remains a hypothesis only. Pradhan *et al*.[19] treated 28 cases of OCD with a BT package and good results were seen in 15 (53%) of them. Therapeutic procedures included relaxation training, thought stopping, implosion, modeling, response prevention, electrical aversion and positive reinforcement. Psychological tests like Hamilton Anxiety Rating Scale, Rorschach Ink Blot test and MMPI were administered before and after treatment. Improvement with BT was not reflected in the post-treatment performance on these tests. Shorter duration of illness was related to better outcome. No information was given on the effect of the package on obsessive and compulsive symptoms separately or on concomitant use of medication. On follow-up, four months to two years later, 27% of those who had good outcome had relapsed. In a case report Singh *et al*.[20] describe use of BT techniques like thought habituation and exposure, along with pharmacotherapy (fluoxetine and thyroxine) in treating a 21-year-old male who presented with obsessive slowness. The symptoms improved in three months and remained so at nine-month follow-up.

### Writer’s cramp

Fernandez[21] reported 17 cases of occupational neurosis, including 13 cases of writer’s cramp. These patients were treated with various methods like narcoanalysis, methedrine abreaction, hypnosis etc. “Prevention of habit formation by daily supervision and correction of handwriting style” also was part of the treatment. All patients were reported to “have improved sufficiently to adjust satisfactorily to their normal life”. The Arora *et al*.[22] paper published in the Journal of Behavior Therapy and Experimental Psychiatry was the most influential paper on BT of writer’s cramp. All subsequent studies from India have used the procedure described in that paper. Mehta *et al*.[23] brought out a large series of 30 cases of writer’s cramp treated with BT consisting of relaxation, retraining and systematic desensitization (in some cases). Complete improvement occurred in 13 patients and partial improvement in 14. Good improvement was associated with good motivation, regular treatment sessions, insight in to the psychological nature of the illness and shorter duration. Another paper on three patients by John *et al*.[24] also reported good improvement in all three patients with supinator retraining. Chavan *et al*.[25] reported a series of 23 cases of writer’s cramp with BT, individual psychotherapy and anxiolytic drugs. BT included JPMR and retraining exercises. Eight patients had only BT, four had BT and drugs, two had JPMR only, five had JPMR and drugs and two had only drugs. Only four patients showed good improvement; eight had no improvement at all. BT as treatment, short duration of illness, long duration of treatment involving frequent sessions were found to be factors associated with good outcome. Though these patients are reported to have had individual psychotherapy, no information is given about the indication for it and its influence on the outcome.

### Anxiety neurosis

Biofeedback, which is often considered a BT technique, was used in the treatment of anxiety neurosis by Sargunraj *et al*.[26] Thirty six patients with a diagnosis of anxiety neurosis were given EMG biofeedback. Twelve of them were also on adjuvant medication. Biofeedback training resulted in lowered levels of EMG activity during mid and post-therapy assessments, but there were no concomitant changes in skin temperature, skin conductance level and response. Anxiety symptom score also decreased, but there is no mention of the clinical significance of this reduction.

Sahasi *et al*.[27] compared the effect of JPMR with yogic techniques of relaxation in the management of anxiety neurosis. Both groups showed significant reduction in anxiety levels, but greater improvement in state anxiety happened in the yoga group. Subjective improvement also was more in the yoga group. One drawback of this study was that the therapists themselves were the assessors of outcome. Reduction in symptom scores in the psychological measure of anxiety and self report by the subjects was achieved by EMG biofeedback assisted relaxation in a group of 22 persons with anxiety neurosis by Abraham *et al*.[28] but this was not reflected in physiological measures like GSR.

### Tension headache

Sethi *et al*.[29] compared the efficacy of biofeedback and shavasana in tension headache. Sixteen patients were randomly assigned to two groups after excluding physical cause for headache. Both the groups were treated for 10 weeks. Equal response was seen in both the groups. The authors use this finding to advocate greater use of yoga in the treatment of problems like tension headache, because it will be more effective in a country like India. However, it is to be noted that the sample size was small and the completers were only seven in the shavasana group and six in the EMG group. Only five from both the groups put together showed complete remission.

Five executives with tension headache were treated by Mehta[30] with JPMR supplemented by relaxation practice at home, and brief relaxation and cue relaxation during office hours. All five showed marked improvement both according to subjective report and daily headache diary.

Gada[31] compared the effectiveness of EMG biofeedback and JPMR in tension headache. Peak headache intensity, average daily headache activity score and headache free days were used as outcome measures. Both methods were found to be significantly effective. The study highlights the appropriate use of a cost effective procedure like JPMR.

### Trichotillomania

Trivedi *et al*.[32] reported a case of trichotillomania initially treated with anti depressant medication and anxiolytics and then given psychotherapy, which improved his insight but did not reduce the urge to pull the hair. Then he was given BT, which involved bandaging both his hands for long hours to prevent hair pulling, making the patient to listen to music, reading books etc, as diversion from the repetitive behavior and positive reinforcement for refraining from hair pulling. Three weeks’ of treatment resulted in good control of the habit and this was maintained at three months’ follow-up.

In a more recent paper, Kaur *et al*.[33] reported three cases of trichotillomania treated with pharmacotherapy and BT consisting of JPMR, deep breathing exercise, distraction technique, response prevention, thought stopping and diary maintenance. Improvement occurred in all cases in three to four weeks. When followed-up six to seven months later, one patient was found to have a mild relapse which was controlled by further BT. Despite the impressive results, the application of far too many behavioral techniques makes it difficult to use them in ordinary clinical practice. The reader is left wondering, which of these techniques is the real effective ingredient!

### “Hysteria”

Seven females diagnosed with ‘hysterical vomiting’ were treated by Bhattacharya *et al*.[34] with electrical aversion, not only for vomiting during treatment session, but also for reported vomiting at home between sessions! All subjects stopped having the symptom. Authors refer to the controversy regarding the use of aversive techniques as treatment, but feel they are justified in resorting to them.

Vyas *et al*.[35] reported the use of aversion therapy in ‘hysterical fits’. Thirty six patients were treated in two phases – First phase of inducing fits and second phase of applying aversive stimuli till the fits stopped. All reported to have been “cured” at the end of the treatment phase and 72.33% did not have any fit at follow-up six months later. The authors’ conclusion, “aversion therapy is as good as any other mode of treatment for hysterical fits. It is less time consuming than other conventional psychotherpeutic and psychoanalytical procedures,” may not be an adequate justification for its continued use in dissociative and similar disorders.

Nasirabadi *et al*.[36] reported about a young man who presented with seven episodes of hysterical aphonia in ten years. Each time he was brought to the hospital, faradic stimuli were given with electrodes placed on the throat and immediately patient would become symptom-free. It is stated that the patient’s family wanted only symptom removal and did not want any therapy for the underlying psychological problems, perhaps indicating the undesirability of merely relying on the power of suggestion to remove symptoms.

### Alcoholism

Forty eight alcoholics were treated by Bagadia *et al*.[37] with electrical aversion in groups of three, when one patient got the aversive electrical stimuli, the other two were expected to observe him. Each patient had a minimum of 20 aversion sessions. Outcome measures included not only abstinence, but also work, social behavior, reduction in daily consumption of alcohol, reduction in drunken days per week and reduction in urge to drink. Patients were followed up for six months to two years; 60.4% showed good improvement. Instead of confining to abstinence alone as an outcome measure, making use of other clinically significant measures of outcome is the strength of this paper. Details about concomitant pharmacotherapy are not given and their possible contribution to outcome not discussed. The rationale of making two patients watch the third one get aversion therapy is also not explained.

### Depression

Ten patients diagnosed to have ‘neurotic depression’ were given multi-modal BT by Kumariah.[38] Treatment included measures to increase activity level, reduce behavioral excesses, induct affects that are incompatible with depression and enhance instrumental skills given in18 to 31 sessions. Dysphoria and somatic complaints disappeared, behavioral excesses and deficits came down to optimum levels and patients described themselves to be “happy at work and in life” No rating scale for depression was used at baseline or follow-up.

### Miscellaneous conditions treated with behavior therapy

The earliest case report on the use of BT, published in IJP was by Ratan Singh[39] who treated a 26-year-old man who used to tear and bite his nails and skin of finger tips almost continuously except when he was asleep. Initially the patient was treated with negative practice. Though the habit was controlled for some time, it recurred when the patient went back to his college. In the next phase of treatment, antecedents of the habit were identified as situations which made him anxious and apprehensive. JPMR was introduced and patient was asked to practice it in situations which made him bite the nails. This led to a remarkable improvement which was maintained at four months’ follow-up.

Nammalvar *et al*.[40] treated 29 patients with assorted symptoms like aphonia, titubation, tremors, and belching of four to six months’ duration. Electrical aversion was given for maladaptive behavior; shock was terminated when adaptive behavior occurred, which was also socially reinforced. Behavioral counseling was given to family members to reinforce adaptive behavior. Twenty five of the 29 persons recovered from the symptoms and remained so at follow-up nine to 14 months later.

A four-and-a-half year old child with an I.Q of 110, with encopresis for two and a half years, unable to even attend nursery school, was treated by Behre *et al*.[41] Basal rate of the problem behavior was established. Then positive reinforcement was introduced to promote successful elimination in to the toilet. If no bowel movement occurred no reward or punishment was given. Mother was required to give the reward as well as to chart the period of dryness. Improvement started after two weeks of therapy and in six months the child had completely stopped soiling himself. Inspite of being a single case report, this deserves attention because of the systematic way in which operant conditioning was introduced to eliminate a clinically relevant problem.

Reevar[42] reported behavioral management of a case of ‘hypochondriasis’ with JPMR, behavioral counseling, and bibliotherapy. Clinical details given in the report raise doubts about the diagnosis and description of ‘behavioral counseling’ and ‘bibliotherapy’ are quite different from what is generally understood by these terms.

### Advent of cognitive behavior therapy

The emergence of Cognitive Therapy (CT) as an effective treatment approach for many conditions like depression and anxiety disorders and its subsequent merger with BT to form Cognitive Behavior Therapy (CBT) has had its impact on the Indian scene also. There are a few psychiatrists and many psychologists practicing it, but publications in this field in IJP have been few. An early paper by Kuruvilla[42] on 17 patients with major depressive disorder showed that CBT can be practiced in Indian setting also. Of the 14 patients who completed the course, 11 showed marked improvement and three had partial improvement in depressive symptoms. Like any psychological method of treatment CBT also needs some modifications and adaptation to suit the culture in which it is practiced. This issue is dealt with in another paper by Kuruvilla.[43] In a review article on CBT, Kuruvilla[44] traces its origin, theoretical foundations, and early applications in conditions like depression, and anxiety disorders. Its current place in the treatment of psychotic conditions, dysthymia, obsessive compulsive disorder, personality disorders, hypochondriasis, PTSD, alcoholism etc. are summarized with brief mention about efficacy studies in each area.

The few original reports on the use of CBT which have appeared in IJP are reviewed below.

### Cognitive behavior therapy in psychosis

Shriharsh *et al*.[45] treated 51 patients with schizophrenia or schizo-affective disorder with an average of 20 sessions of CBT spread over 10 weeks. Techniques used consisted of psycho-education, behavioral analysis, activity monitoring and scheduling, assertiveness training, relaxation, distraction techniques, systematic desensitization *in vitro*, exposure and response prevention, stress inoculation, skills training and cognitive restructuring. Treatment led to improved adjustment scores in Bell’s Adjustment Inventory, decrease in intensity of perceived symptoms and automatic thoughts. During follow-up, gradual decline in improvement with CBT happened, but at nine months the patients were still better than they were before CBT. The paper does not give information on concomitant anti-psychotic medication which also could have had an influence on the outcome. Description of the symptoms the patients in this study had suggest that at the time of CBT most of them were in a state of post psychotic depression and were not actively psychotic. It is also not clear how many patients have had each of the various interventions listed and their effect on specific problems. Clinical significance of the improvement seen on rating scales is not given either.

In a case report on a 31-year-old man with paranoid schizophrenia, who had the delusion of being controlled, being made to laugh, cry etc. through the internet, Dugal *et al*.[46] report the use of verbal challenge of the delusion, arranging experiments to test patient’s belief and encourage finding alternative explanations. The patient was also on 250 mg/day of clozapine. The delusion disappeared with these interventions and did not recur during the next two months.

### Panic disorder

Thirty patients with panic disorder were treated by Manjula *et al*.[47] in two groups of 15 each. One group was treated by CBT consisting of psycho-education, applied relaxation, cognitive restructuring, interoceptive exposure and *in vivo* exposure. The second group was treated by what the authors call ‘behavioral intervention’ (BI), which included only psycho-education and applied relaxation. BT group had 15 to 20 sessions in five weeks while the BI group had eight to 10 sessions in two weeks. Although both the groups showed improvement, the CBT group was superior in the reduction of panic symptoms, avoidance behavior, safety behaviors and negative cognitions. In a large percentage of CBT patients the magnitude of change was clinically significant. The therapist herself being the assessor of improvement, inequality in the time of contact with the therapist as well as total duration of treatment are some the methodological problems that could have influenced the outcome. The BI group had only psycho-education and applied relaxation as its components whereas standard BT of panic disorder also include interoceptive exposure and *in vivo* exposure while in this study they are part of CBT only.

## CONCLUSION

In a survey of the current scene on the use of CBT, Kuruvilla[48] evaluates the evidence base to support the use of CBT in a number of psychiatric disorders, its role in the management of certain physical disorders, innovations in the delivery of CBT and the current findings which show that CBT modulates the functioning of specific sites in the limbic and cortical regions of brain. These findings support the conclusion which Prochaska and Norcross[49] arrived at after evaluating various psychotherapies, “Cognitive Behavioral approach is the fastest growing and heavily researched psychotherapy in the cotemporary scene”. It will be immensely beneficial for patients in India if psychiatrists in India make greater use of this model of therapy.
